# Towards a dialogical and progressive educational policy framework: Manoeuvring a middle way among the polarised constructs

**DOI:** 10.12688/f1000research.127762.1

**Published:** 2023-01-19

**Authors:** Solomon Arulraj David

**Affiliations:** 1 Faculty of Education, The British University in Dubai, Dubai, 345015, United Arab Emirates

**Keywords:** Public and Social Policy, Educational Policy, Policy Polars and Centre, Dialogical Approach, Progressive Policy Framework

## Abstract

Policy science and practice around the world, including educational policies, are dominated by popular, extreme approaches such as market-orientated approaches at one end and critical argumentative approaches at the other end. This study therefore aims to manoeuvre a middle way to propose a dialogical and progressive educational policy framework and explores the research question: ‘how could a middle way (a dialogical and progressive framework) be manoeuvred among the polarised policy constructs?’ The study embraces Lynham’s five phases of theory building as the basis for this research, which includes conceptual development, operationalisation, confirmation/disconfirmation, application, and continuous refinement. The study explores some of the known existing policy frameworks for conceptual mapping, investigates the underlying dynamics and discourses to operationalise, uses diverse arguments in the literature to confirm/disconfirm and proposes to mark the emerging patterns, trends, and gaps in policy research to apply and refine. The study contends that if it is possible to have a polarised market-oriented and critical argumentative policy frameworks, it is then possible to have a dialogical, progressive middle-way policy framework. The study had to limit to the most important and related theories, and models to focus. Future works could explore a wide range of other relevant theories and models to further investigate this framework. Furthermore, application of the proposed dialogical, progressive educational policy framework in specific context/case may help to refine it. The study contends that the proposed middle way is not a perfect space but a potential space in which a dialogical and progressive educational policy may thrive.

## Introduction

There are a number of approaches to policy design, dissemination, and evaluation. However, public policies including educational policies across the world increasingly embrace the emergence of neo-managerialism that favours neo-liberal, market driven policy frameworks ( David, 2014). Such trends are often countered by radical critical approaches, such as argumentative policy frameworks. It is argued that the growing libertarianism favours laissez-faire capitalism where the market strongly influences public and social policy making and analysis processes. This seemingly leads to the antagonism between agency/ structure, individual/institution, public/ state, and market/state ( Berkhout & Wielemans, 1999).

Education and educational policy have strongly embraced the dominant trends in the world outside education sector making it conducive, while often missing exploring potential alternatives. It is argued that all policy frameworks may have some strengths and some limitations. This study believes that the discontentment of the neo-managerial policy orientation and the polarised critical alternatives create a space for the search of a progressive policy framework. And it considers a dialogical progressive policy framework to stimulate dialogue among polarised frameworks as a middle way. The study therefore aims to manoeuvre a middle way to propose a dialogical and progressive educational policy framework. The polar (north/south or east/west) in this study is ideological rather than geographical. The study streams away from the classical empirical approach and embraces rationalist and hermeneutic approaches by critically reviewing and interpreting relevant literature and making essential meaning by careful reflection and rationalisation.

This research follows Lynham’s five phases of theory building as elaborated in the methodology. The study particularly takes the courage to look for a progressive alternative among existing policy polars. It further explores some of the theoretical blind spots, particularly looking at spaces where conventional theorists seldom visit. The study also explores some of the alternative cognitive models such as the epistemic third space ( Seremani & Clegg, 2015), cognitive justice ( Visvanathan, 2019) and others as potential middle way among the polarised constructs. The study contents that if it is possible to have polarised market oriented and critical argumentative policy frameworks, it is then possible to have a dialogical, progressive middle way policy framework.

## Methodology

The study aimed to manoeuvre a middle way to propose a dialogical and progressive educational policy framework amidst the polarised constructs. Exploration of the relevant theories, models and literature supported the formulation of the general research question relevant to the focus of the study: ‘how could a middle way (a dialogical and progressive framework) be manoeuvred among the polarised (new-liberal/managerial vs argumentative/critical frameworks) policy constructs? The general method of theory building in applied disciplines approach by Lynham (2002) is used as the methodological basis in this study in search of answers and evidence for the above research question. This study follows the first three of the five phases of Lynham’s theory building such as conceptual development, operationalisation, confirmation/disconfirmation, application, and continuous refinement. The last two phases; application and refinement could be extended when this framework is applied in a specific case or context. This research began by mapping out some of the dominant existing policy frameworks for conceptual development. The study then explored the underlying dynamics, cross-cutting discourses using discourse analysis to operationalise the framework. It accounted for diverse arguments in the scholarly world and used a constant comparison approach to confirm and disconfirm. Further, it highlighted the emerging patterns and trends for application and continuous refinement through critical reflection, interpretation, and rationalisation.

Standard protocols of literature review, meta-analysis, and meta-synthesis were followed in the study ( Xiao & Watson, 2017). Relevant literature was searched using relevant search tools and words. Suitable academic databases such as ERIC, EDSCO and journals on public and educational policies such as Public Administration Review, Journal of Comparative Policy Analysis, Review of Policy Research, Educational Policy, Educational Evaluation and Policy Analysis and others were supportive as information sources in search of comprehensive and relevant literature well situated in the field of the study. The study used inclusion/eligible criteria suitable to the nature of the study, particularly sorting theories, models, and literature on three policy polars such as the ‘extreme right’, ‘extreme left’ and ‘the centre’ to select relevant literature for review. The selected literature became the resources and data for further analysis. The use of skimming and scanning helped to sort out recent related literature for further in-depth review. For Aguinis *et al.* (2011), meta-analysis is a central method for knowledge accumulation in many scientific fields. Qualitative meta-analysis focusing on the conceptual focus of the study helped to achieve a comprehensive understanding ( Hansen, Steinmetz & Block, 2022). Meta-synthesis was supportive in summarising, analysing, interpreting the findings. Erwin *et al.* (2011) consider meta-synthesis as a coherent approach to analyse and report qualitative data. This study particularly embraced integrative approach ( Synder, 2019) in summarising and synthesising the review to reach a comprehensive meaning making. The study also relied on constant comparison and thick descriptions among related and polarised policy frameworks to analyse, interpret, and synthesise ( Creswell, 2007) to develop the proposed middle way ‘dialogical and progressive educational policy framework’.

## Review and analysis

**Conceptual development** is the first phase of theory building for Lynham, which “requires that the theorist formulate initial ideas in a way that depicts current, best, most informed understanding and explanation of the phenomenon, issue, or problem in the relevant world context (2002, p.231)”. Glaser & Strauss (1967) suggest that conceptual thinking and theory building is interrelated with the qualitative research method approach ‘grounded theory’. Some of the pressing questions while conceptualising public and educational policies are: Why do we need policy? What should a policy address? Who designs policy? For whose interest? Who should be involved in policy processes? Who and what should be consulted in policy making, implementing and evaluation? What are the general trends, patterns, procedures, gaps in policy making and analysing? These and other related questions on policy making, implementation and evaluation may help in making a good conceptual development on educational policy making, implementation and evaluation.

Defining public policy would be the foundation for establishing a good conceptual understanding. Public policy is a set of government decisions with a certain common purpose for the public. Birkland (2005) highlights the following as common traits of public policy. For him, public policy is made in the name of the public; and it is generally made or initiated by government, it is interpreted and implemented by public and private actors, it is what the government intends to do, and it is what the government chooses not to do. The term policy has range of definitions. It is often used in synonym with other terms such as principles, rules, and guidelines. According to Compliance Dictionary (2022), policies are formulated or adopted by an organisation to reach its long-term goals and it is usually published in a booklet or in another form that is widely accessible by all. Public policy is the laws and regulations that are made by legislative statesmen and implemented by public administration personnel ( Wu & He, 2009). Most often, the terms ‘policy and law’ are used interchangeably. It is necessary to clarify the differences between the two terms. According to Education and Training Unit (2018), a policy lists what a government ministry wants to achieve and the methods and principles it will instrument to attain them, while laws set out standards, procedures, and principles that must be followed. A policy document is not a law, but it will often identify new laws needed to achieve its goals. And laws must be guided by current government policies. It is important to observe the gap between the intention and the outcome of a policy. Qian & Walker (2011) indicate the gap between the policy intent and policy effect of the curriculum. Often, the end users’ experience of a policy is far from the intentions embedded during the design ( David & Hill, 2020). In furthering the conceptual development on policy making and evaluation, Haddad’s ( 1995) conceptual framework for policy making and analysis is worth mentioning, as that includes “analysis of the existing situation, the process of generating policy options, evaluation of policy options, making the policy decision, planning policy implementation, policy impact assessment, subsequent policy cycle (p.18)”. The following table offers the scope of policymaking, implementation, and evaluation, adapted from Haddad (1995, p.19).

The above table offers necessary foundation for the conceptual development. The framework indicates that if the issue is high in a global context, complex and diverse programmes would be favourable with broad and universal strategies. For Haddad (1995), the scope of policymaking, implementation and evaluation must consider the issue, programme, and strategies. The issues could be low, precise, and narrow. The programme can be complex, with several alternatives and criteria along with the decision environment, while the strategies could be high, imprecise, and broad. If the issue is medium and the context is regional, dynamic and several programmes are suitable with narrow and priority strategies. And if the issue is low and the context is local, specific and few programmes are relevant with precise and focused strategies. The choices of the programmes and strategies may change if the issues and contexts are different as indicated in Table 1. Policy is a broad notion including any kind of management activity. The term ‘policy’ as such defines the art of the state management. Policy means authority and decision-making. Thus, policy is referred to as the management of an activity or influence on such management. Many things in the world today have largely been shifted by globalisation phenomenon, which impacts the policy processes ( David, 2014). However, the changes induced by globalisation may not happen the same way across the world. Countries and activities in different countries differ in responding to the process of globalisation according to their capacities and preferences. Yet, they are increasingly governed by similar pressures, procedures, and organisational patterns ( Schugurensky, 1990). This reality is experienced at all levels of governance and at all levels of social sectors. The way, in which the economy, politics, culture were organised at supranational, national, and sub-national level has changed during the globalisation regime ( Wielemans, 2002) and these changes have implications for policy processes.

**Table 1.** Scope of policymaking, implementation and evaluation (Adapted from Haddads’, 1995).

Issue specific	Programmes	Strategies	Context
High	Complex/diverse	Broad/universal	Global
Medium	Dynamic/several	Narrow/priority	Regional
Low	Specific/few	Precise/focused	Local
High	Complex/several	Broad/priority	Regional
Medium	Dynamic/few	Narrow/focused	Local
Low	Specific/diverse	Precise/universal	Global
High	Complex/few	Broad/focused	Local
Medium	Dynamic/diverse	Narrow/universal	Global
Low	Specific/several	Precise/priority	Regional

**Figure 1.**
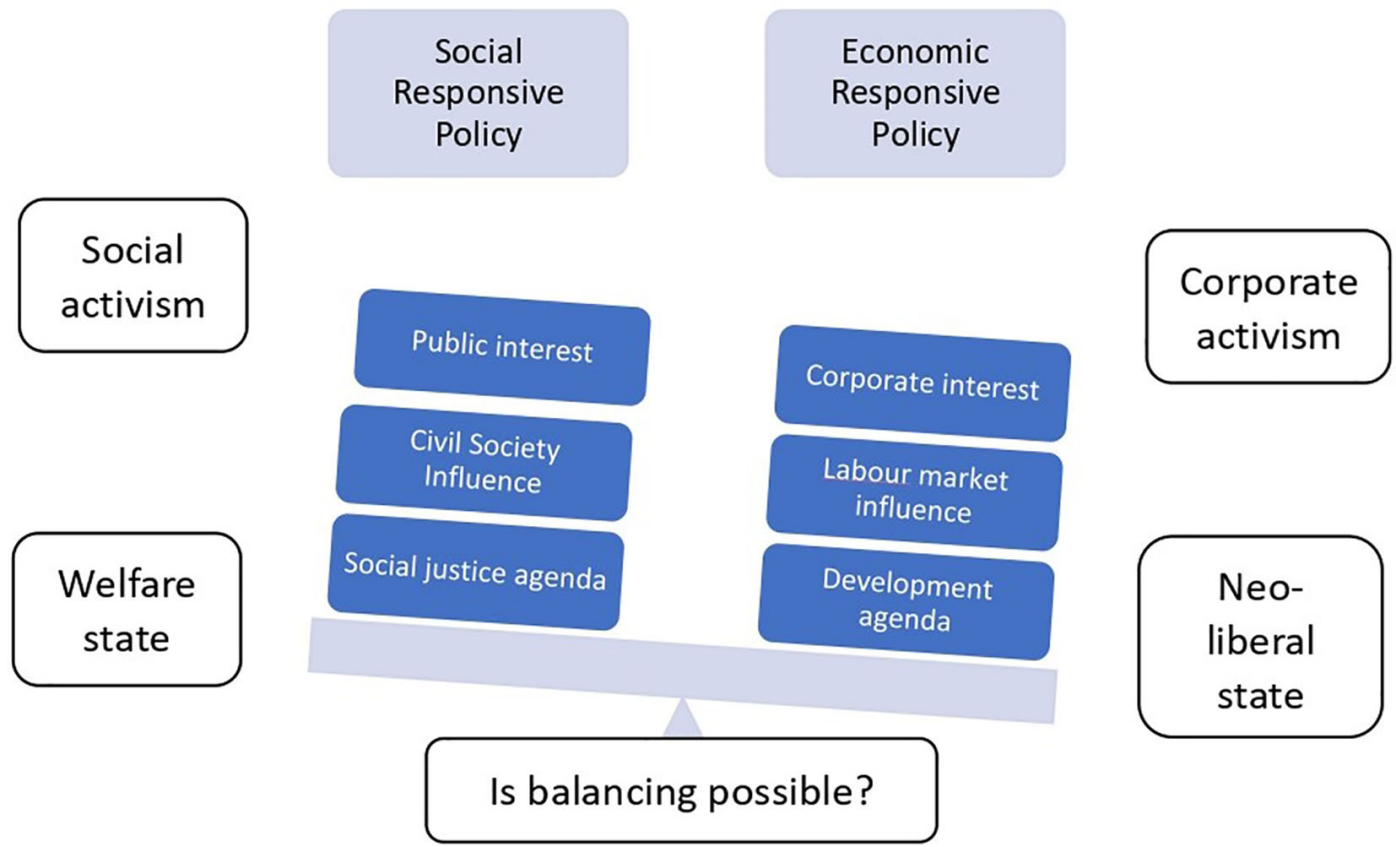
Conceptualisation of polarised policy arena.

Many scholars including Burbules & Torres (2000) elaborate on the way globalisation is impacting education policies of nation-states. They indicate the supranational institutions’ influence on national policies (with a set of global rules), the impact of neo-liberalism as a hegemonic policy discourse, reflected in policy making at different levels ( David, 2014). For some public policy is firmly held in the grip of the free market principles (profit as the ultimate goal) ( Cerny & Evans, 2004). Moreover, economic forces, labour market requirements exercise considerable impacts on public decision making. This paves the way for public and educational policies to be measured in terms of its capacity to be useful and to serve the labour market ( Wielemans, 2000, David & Wildemeersch, 2014). It is important to note that the free market economy only works adequately if certain conditions are met (with due economic return). Thus, the strong dependency of public policy making, with free market economic principles might damage public and social interest ( David, 2017a). In this conceptualisation, it is important to notice the polarised constructs in the existing policy arena. The polarised constructs are essential for the proposed progressive framework (middle way) to thrive. An important question at this stage is: ‘Is balancing possible?’ assuming that the two polarised tendencies in policy-making are social and economic responsiveness. For which, accounting for different stakeholders’ views are essential to understand the policy arena ( David, 2017b). The Figure 1 is conceptualising the polarised policy arena with the two polars such as the social responsive end and the economic responsive end.

The above conceptualisation of the polarised policy arena hints the two extreme ends of the policy space that are led by social responsive end on one side and economic responsive end on the other side. The social responsive end is driven by social activism and governed by welfare states, while the economic responsive end is driven by corporate activism and governed by neo-liberal states ( David, 2016). The social responsive policy is measured by its focus on public interest and social justice agenda, influenced by civil societies, while the economic responsive policy is measured by corporate interest and development agenda, influenced by the global and local market ( Rensburg, Motala & David, 2016). As this polarised conceptualisation compels the question, is balancing possible? The potential answer would be that it is possible, if a middle and progressive way is explored. Therefore, the fundamental assumption of this study is that if it is possible for us to have the polarised neo-managerial, market oriented and critical, argumentative policy frameworks, it should be then possible to have a dialogical, progressive middle way policy framework. In such conceptualisation a dialogical progressive middle way policy framework is explored.

**Figure 2.**
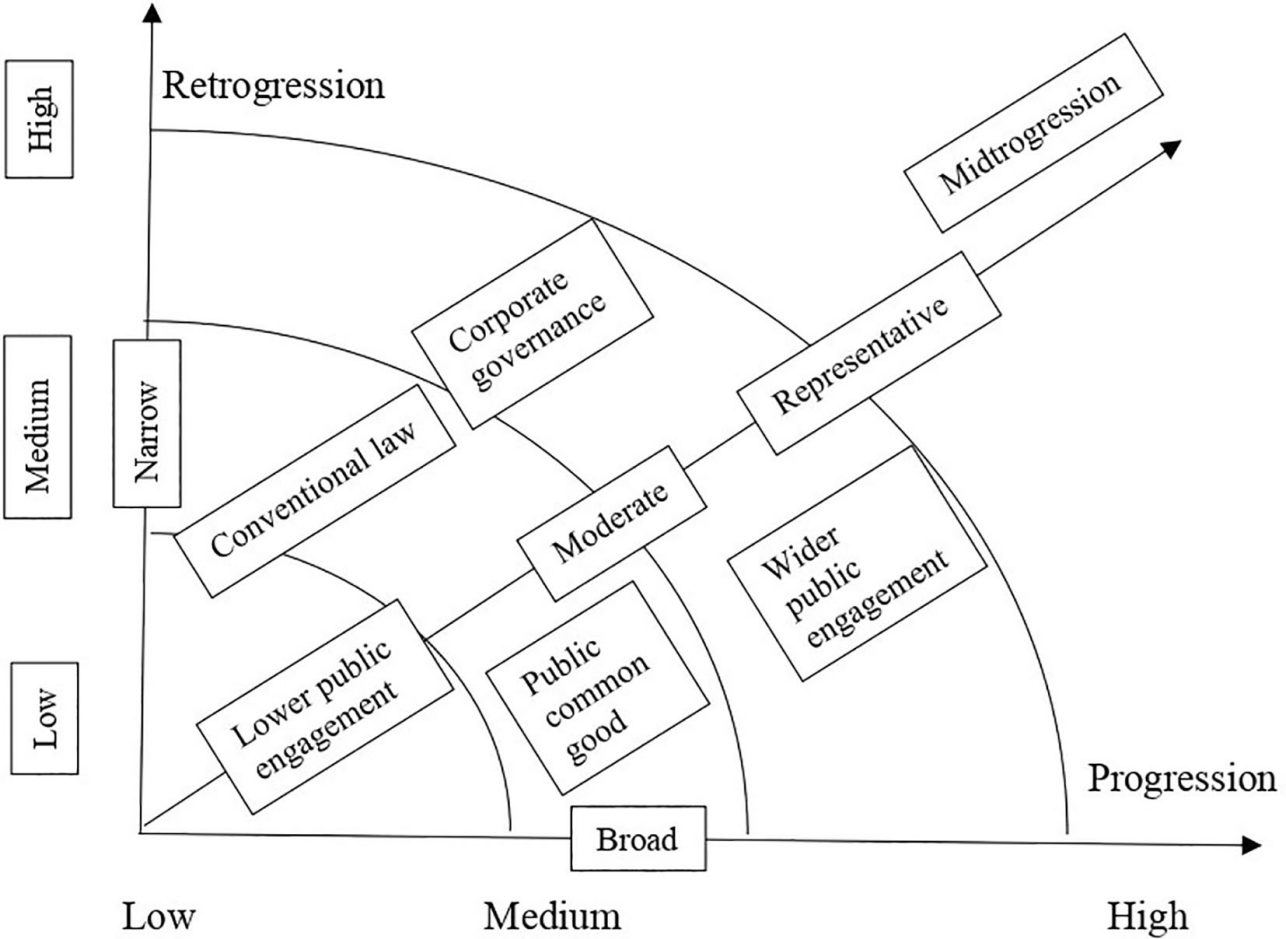
Conducive environment for progressive policy.

**Operationalisation** is the second phase of theory building for Lynham (2002). For her, the purpose of operationalisation phase of theory building research is essentially an explicit connection between the conceptualisation phase and practice. Understanding public policy in light Durkheim’s (1968) construct of collective or common consciousness is important in looking for a progressive policy environment. For him, a good society is based on a moral order and the moral order is achieved through collective consciousness, as society commands us because it is exterior and superior to us. He sees social and public policy as a social/group decision making, which is a cult of a collective consciousness process. However, it is important to distinguish group decision making from groupthink. Lunenburg (2010) differentiates group decision making from groupthink, that group decision making is the tendency of cohesive groups to reach a consensus on issues without offering, seeking, or considering alternative viewpoints, while groupthink ( Janis, 1991) is a result of group pressures to reach a consensus which might lead to a deterioration of mental efficiency, poor tasting of reality and lack of moral judgement for individuals. As policy making involves decision making, Etzioni (1967) recommends mixed scanning approach for social decision making. The rationalist approach is utopian and as incremental approach lacks empirical facts. Therefore the mixed scanning approach that makes detailed search for higher order fundamental decisions. In order to operationalise the dialogical progressive policy framework, it is necessary to further theorise this framework. Exploring some of the established theories to argue the need and relevance for a public policy becomes fundamental. The Hobbesian problem of social order ( Ellis, 1971) and social norms ( Hampton, 1987), Kant’s social contract ( Stanford, 2016) insist on the need for a social and public policy that would stabilise social order. They viewed that the social order could be established by the formation of the state where individuals’ compliance to social norms would create social order. Marx & Engels’ view on ‘state as a necessary institution for social order’ further poses the need for social and public policy to be facilitated by a social institution such as the state ( Sanderson, 1963). For them the state as a social institution could manage class conflict and with class compliance with social norms would create social order. Social institutionalism legitimises that the social and cultural norms are essential for public management and social and public policy making. Sceptics such as Proudhon were not convinced that the state could be an absolute end for social order. Proudhon’s list of the ‘domestic inconveniences of the state’ indicates the states’ limitations in establishing social order, who further recommends ‘self-government and the citizen state’ ( Panarchy, 2019), Foucault’s notions, such as bio power and social control ( Lacombe, 1996), recommend controlling and disciplining society by exercising power to normalise society. On the contrary, Noam Chomsky argued that social change and norms must be founded on some concepts of human nature and freedom ( Wilkin, 1999). Buchanan & Tullock’s public choice theory ( Holcombe, 1989) highlights that the political behaviour of citizens in the public choices they make influence on the political processes and outcomes that are essentially important in understanding and operationalising the progressive policy framework. Baumer & Van Horn’s (2014) policy making spectrum includes three types of policy decisions such as narrow/private, moderate/representative democracy and broad/public. The narrow decision is made by corporate governance and capitalism, the moderate decision is made by elected candidates and conventional law making, the broad decision is made by elections and aroused public opinion. In such understanding of public policy making and decision-making processes, a conducive environment for progressive policy is speculated. The following framework presents a conducive environment for progressive policy ( Figure 2).

The above framework on conducive environment for progressive policy envisions a space in which a progressive policy making could thrive. Progression is viewed as a broad policy making type, which considers wider public engagement, while retrogression is viewed as a narrow policy making type that relies on mere conventional law and midtrogression is a moderate policy making type, which is representative. Each of these three types could apply their approaches in low, medium, and high levels. The high progressive end is the ideal conducive environment for progressive policy, with public common good and with wider public engagement. Following the development of the conducive environment for progressive policy making the framework for dialogical and progressive framework is designed. The proposed ‘dialogical and progressive public and educational policy framework’ is partly drawn from Mitchell’s (2018) progressivism and the evolution of education policy. His classification of progressivism includes emphasis on structural reforms, policy shift toward access and participation, shifting away from centralised control and accounting for change. The proposed framework investigates the frameworks of Lejano (2006) that provides the evolution of policy analysis framework from positivist foundations, post-positivist’s perspectives, and post-constructivist’s sentiments. As Peters (2005) indicates that it is important to understand the policy problem well before further conceptualising the design, tool and strategies, the design makes necessary attention to ranges of approaches to policy making theories while designing the proposed dialogical, progressive policy framework.

**Figure 3.**
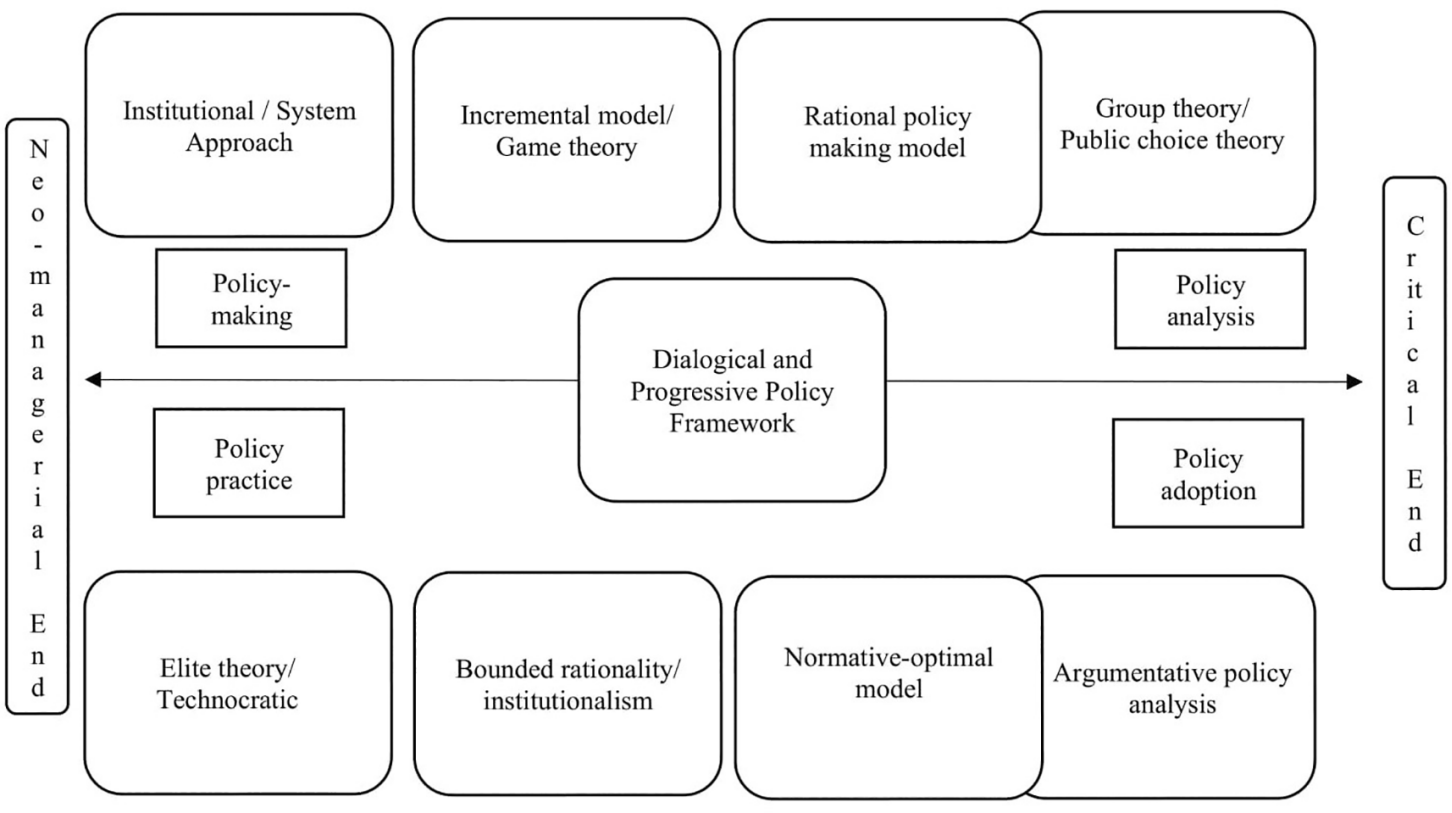
Dialogical, progressive policy framework.

The proposed ‘dialogical and progressive policy framework’ is partly drawn from John’s (2011) idea of making policy work, in which he considers information, persuasion and deliberation as key to make a policy work better. He considers the following as crucial to achieve this; public information campaigns, persuading the citizens directly, smart information provision, using social pressure, social support, thinking, not just nudging, and educating. Fowler (2004) offers evaluation tool to determine if the policy works. He insists that evaluation is an integral part of policy process, evaluation must be professional, there should be good purposes for evaluation, there must be good criteria for judging evaluation (such as usefulness, feasibility, propriety (legal/ethical), accuracy), needs to classify evaluation (summative, formative and pseudo), and evaluation process must include goals, indicators, data collection instruments, report, response to evaluator’s recommendations. The Figure 3 presents the dialogical, progressive policy framework.

The above framework on dialogical and progressive policy is drawn as a middle way between the two polarised ends of neo-managerial/technocratic policy at one end and argumentative and critical policy on the other end. These two polarised ends are supported and opposed by different theories, models, and practical implications, throughout the policy processes such as the planning, implementation, evaluation, and adoption ( David & Motala, 2017). It is necessary to also acknowledge that the centralised policies do as well have discrepancies ( Mavrogordato & White, 2017). In establishing such dialogical and progressive policy framework, it is necessary to consider a range of theories and models in public policy studies. Chakrabarty & Chand (2016) recommend a range of theories and models for consideration, such as; institutional approach (traditional tool of studying political activities of the government), group theory (group interest and attitude are influential factors in public polices), elite theory (public policy as the interests, preferences and values of the government elite), rational policy-making model (policy-making is a choice among policy alternatives on rational grounds), Simon’s bounded rationality (human rationality is limited and bounded to preferences), incremental model (continuum of the previous government activities), normative-optimal model (which tries to avoid both extremes), game theory (helps understand collective human activity as the outcome of interactive decisions), system approach (public policy as an outcome of political process) and public choice theory (the application of economic analysis on the choices public make). Howlett & Ramesh (2009) offers the levels of analysis in relevant policy related theories. If the unit of analysis is individual, public choice theory fits as a good approach, if the unit of analysis is collective, class analysis, group analysis is favourable, and if the unit of analysis is structure and system, institutionalism fits well.

Peters & Pierre (2006) propose argumentative policy processes (APA) as a constructive tool for public policy analysis. APA includes pathos (a quality that evokes pity and sadness), ethos (the set of beliefs, ideas about the social behaviours), and logos (a principle of order and knowledge). They draw the framework from Toulmin method of argumentation, which includes fact, warrant, backing, conclusion, and rebuttal ( Moxley, 1993). The cost-benefit analysis has been very influential in policymaking and analysis process, largely in the neo-managerial policy processes ( Rensburg, Motala & David, 2015a). Weimer & Vining (2017) indicate that the cost benefit analysis includes the following steps: specify current and alternative policies, costs and benefits count, catalogue relevant impacts, predict impacts, monetize all impacts, discount benefits and costs, compute the present value of net benefits, perform sensitivity analysis. Kraft & Furlong’s (2018) orientations to policy analysis, such as scientific and political is worth exploring. Obviously, each of these orientations have its own strengths and limitations. The Vicker’s Model (1965) of decisional analysis as promoted by Sapru (2017) recommend the consideration of reality judgements (What is out there? What is the problem? What predictions can be made?), value judgements (What values/norms are set? What ought to be?), and action judgements (what to do? How to do it? What actions to take? What solutions are good enough?) to operationalise decision. This theoretical exploration needs to be verified and confirmed with arguments for and against in scholarly world that may further take the proposed model to be tested and validated.

**Confirmation/disconfirmation** is the third phase of theory building for Lynham (2002). This phase falls within the practice component of applied theory building for her. Comparing policy processes would benefit the establishment of rigorous theories and models of policy framework, as constant comparison supports the development of rich descriptions in theory building ( Fram, 2013). As Young & Lewis (2015) hint that consulting similar research on educational policies complements, challenges and sometimes complicates further research, confirming and disconfirming process is not easy. For Creswell (2007) grounded theory research attempts to derive a general, abstract theory of a process, action, or interaction grounded in the views of participants in a study. Creswell considers that the two popular approaches to grounded theory according to him are the systematic procedures of Strauss and Corbin and the constructivist approach of Charmaz. This study particularly applies the constructivist approach of Charmaz. The constructionist approach deals best with what people construct and how this social construction process unfolds ( Charmaz, 2008). Constant comparison and grounded theory approaches are widely used in policy research. Heikkila & Cairney (2017) suggest three criteria for the theoretical approaches to compare the policy process. The first criterion is the extent to which the basic elements of a theory are covered, which includes: a defined scope of analysis, a shared vocabulary/concepts, explicit assumptions, identified relationship among key concepts, and a model of the individual grounding of the theory. The second criterion is the development of research, that covers: how the approach has been employed actively by researchers and published as journal articles and books, how it has been tested, inclusive of diverse policy issues, different political systems and with multiple methods, how the scholars involved in using theory and research, and how the theory has been adapted or modified over time, particularly for the minimum common good for all ( David, 2019).

**Figure 4.**
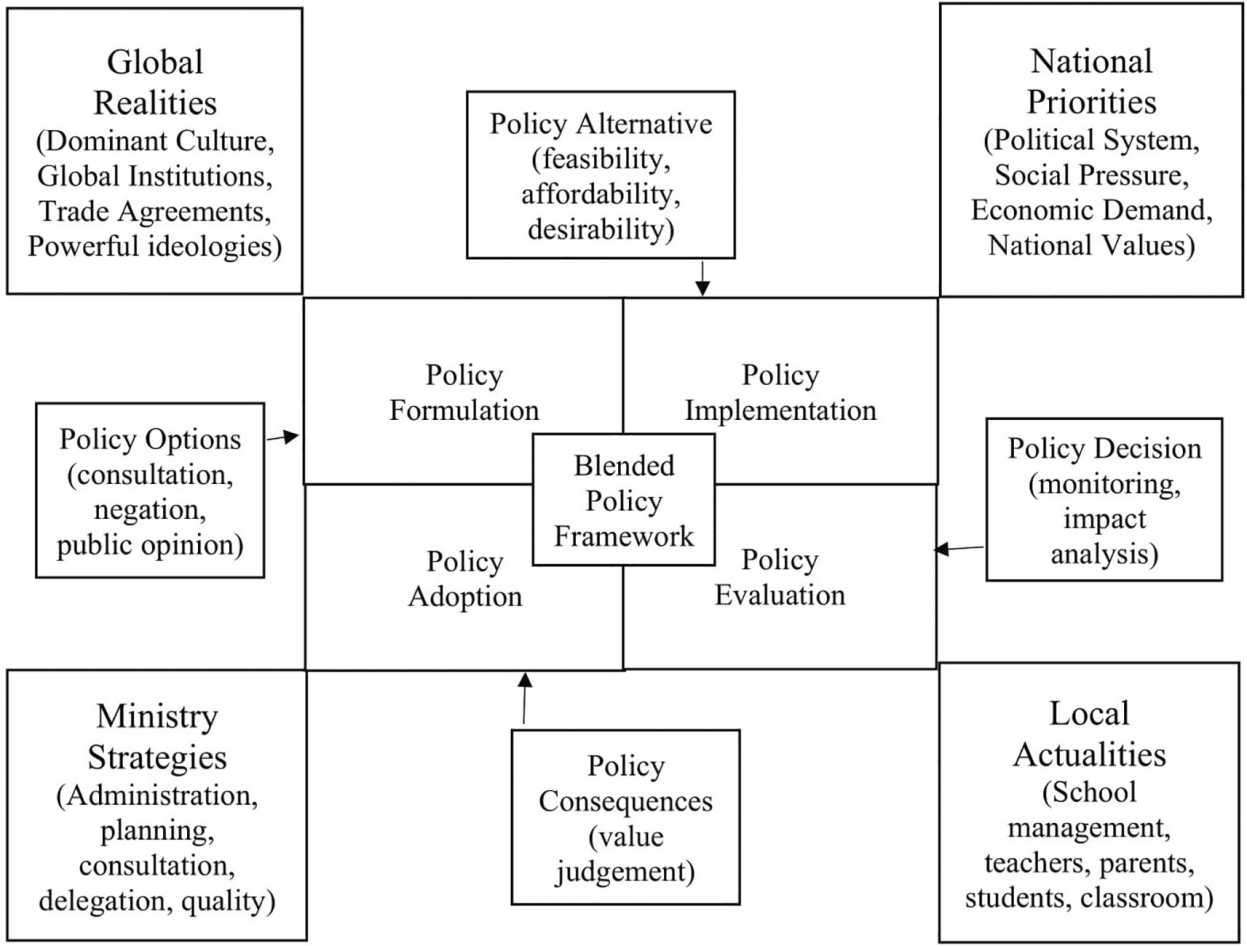
Blended framework for policy processes.

The third criterion is the development of indicators on whether the theory explains a large part of the policy process. It is important to realise that the policy process is complex and there is no general theory. Therefore, they suggest simplifying a complex world to understand it better by asking a fundamental question; which elements do policy scholars treat as crucial to explanation? And they consider the following crucial elements and the interaction between them to provide an overall explanation of the policy making process and systems. These elements include actors, making choices, institutions, networks or subsystems, ideas or beliefs, policy context and events. The complexity of social policy processes depends on the political and managerial processes attached to it. Berkhout & Wielemans’ (1999) integrative approach to education policy indicate that the conceptualisation and understanding of education policy in complex socio-political systems is more problematic than merely as the set of executive, administrative, deliberative, official texts that direct education at the various levels of government. Their integrated approach to educational policy analysis spans from the classroom practices to the broader social systems. The following is a combined approach to public and social policy processes, called the blended progressive dialogical policy framework, which is drawn from several frameworks, particularly from Berkhout & Wielemans’ (1999) integrative approach to education policy and Haddad’s (1995) conceptual framework of policy ( Figure 4).

The above blended framework for policy processes elaborates on the interrelation of policy with complex and dynamic political, social, and managerial processes. The blended policy framework considers the interrelation with global realities, national priorities, ministry or organisational strategies and the local actualities with end users. The framework in interrelating with global realities understands the emergence of dominant culture, the role and advocacy of global institutions, the impacts of trade agreements and the implications of the dominant and powerful political ideologies. In consideration to the national priorities, the framework looks into the political systems in the country, social pressure from different interest groups, the quest for economic growth and development, and the national values. With reference to the ministry or organisational strategies, the framework reviews the level of planning, administration, consultation, delegation, and quality of the policy processes ( David & Abukari, 2019). In the consideration of the local actualities, the framework pays attention to the involvement of teachers, parents, students, educational administrators, and other stakeholders in institutional and classroom management practices. These interrelations at the blended policy framework occurs throughout with policy processes such as policy formulation, implementation, evaluation, and adoption. The blended policy framework makes careful policy alternatives considering the feasibility, affordability, and desirability ( David & Hill 2021). And the blended policy framework takes into consideration of the policy consequences by value judging the policy processes. Robertson (1998) states that policies may fail either by not achieving their goals or may fail to retain political support.

There are several theories of policy processes. Schlager & Blomquist’s (1996) three dominant political theories of the policy processes, such as Sabatier’s advocacy coalitions framework (ACF), institutional rational choice (IRC), and Moe’s political theory of bureaucracy have gained wide attention. There is a different classification of policy processes. The six criteria of policy processes, according to Schlager & Blomquist (1996), include the boundaries of inquiry, the model of the individual, the roles of information and beliefs in decision making and strategy, the nature and roles of groups, the concept of levels of action, and the ability to explain action at various stages of the policy process. Howlett & Ramesh (2009) classify the stages of policy cycle, which includes agenda setting, policy formulation, decision-making, policy implementation and policy evaluation, and the key actors involved are policy universe, policy sub-system, government decision makers, policy sub-system, policy universe. Evidence based policy that was possibly introduced by Adrian Smith, made remarkable impact on policy studies. In his 1996, presidential address to the Royal Statistical Society urged for a more evidence-based approach commenting that it has valuable lessons to offer (Wikepedia, 2018). Evidence based policy (EBP) is a set of methods which informs the policy process, rather directly affect the eventual goals of the policy. A rational, rigorous, and systematic approach is recommended by EBP. The pursuit of EBP is based on the premise that policy decisions should be better informed by available evidence with rational analysis ( Sutcliffe & Court, 2005). The following are the steps in evidence-based policymaking: programme assessment, budget development, implementation oversight, outcome monitoring, targeted evaluation ( The Pew Charitable Trust & MacArthur Foundation, 2014).

**Figure 5.**
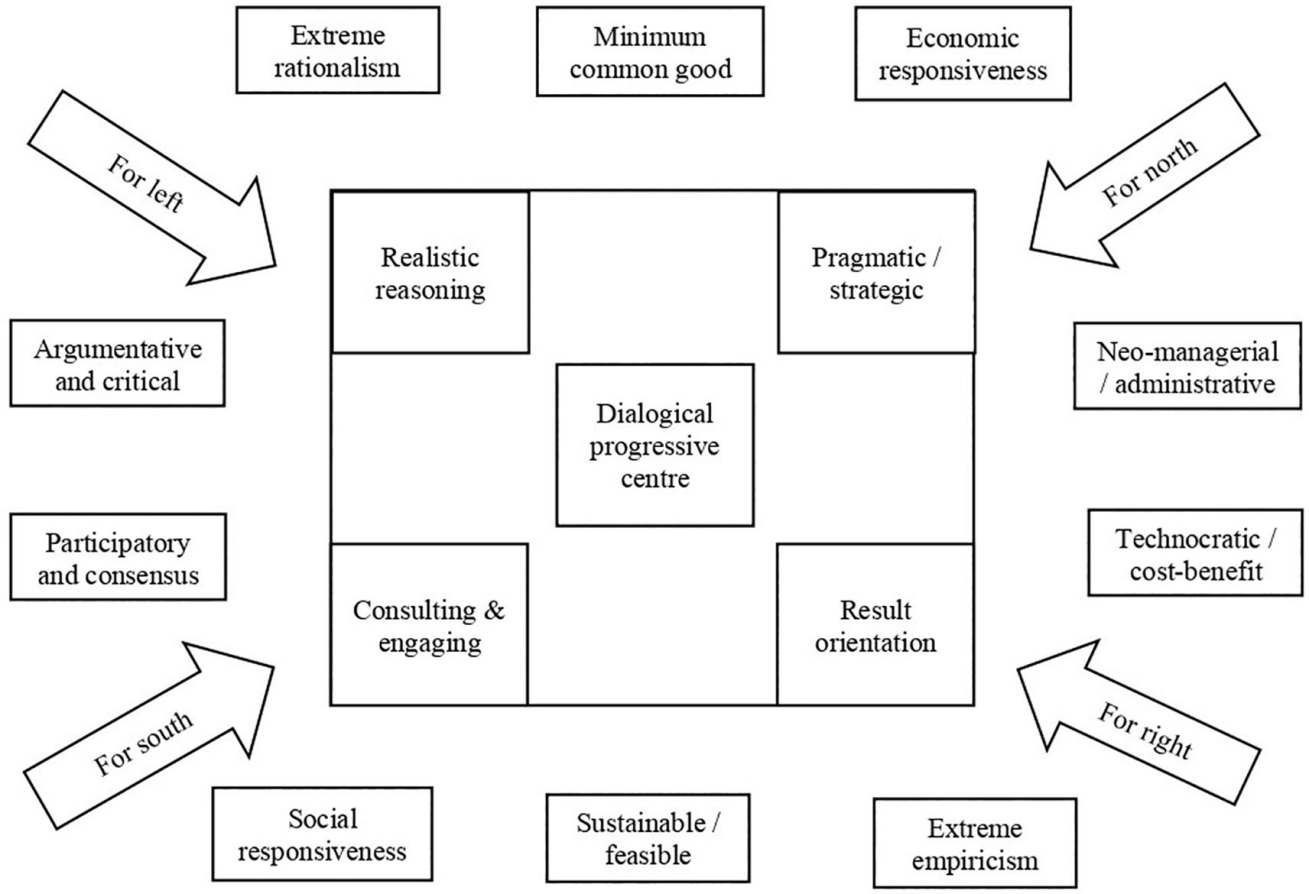
Manoeuvring the middle way.

Explicit policymaking is more a new approach many policy researchers advocate. It is linked to the “explicit rationing or explicit decision-making process referring to decisions made by an administrative authority as to the amounts and types of resources to be made available, eligible populations, and specific rules for allocation. Significant amounts of explicit rationing occur in public and private plans regarding levels of available technology, location of facilities and programmes and expenditure levels. In contrast, the implicit rationing refers to discretionary decisions made by managers, professionals functioning within a fixed budgetary allowance ( Taylor, 2012, Para.4)”. The quality of policy objectives impacts on the policy processes and outcomes. ISO 9001 quality objectives of policy include having measurable objectives, doing gap analysis, having project plans, taking necessary training, documentation, using and improving quality management system, doing internal audits, and ISO registration ( International Organization for Standardisation, 2008). Critical policy analysis is one of the prominent policy analysis tools, several researchers use. Dryzek (2008) considers policy analysis process as a critique. He suggests the following approaches: critique and its opposites – technocracy (identify cause and effect), critique and its politics (technocratic analysis is centre-left, accommodative analysis adjusts to conservative fashion, critical is political ideological free), policy as a science of democracy, policy as progressive democratization of mankind. He considers critical communication, argumentative turn, linguistic turn, the claim to truth, sincerity, comprehensibility, and appropriateness attached to inter-subjective communication as critical standards, which he draws from the critical theory of Habermas. Grover & Rihani (2010) call for humane public policy. They believe that using a complexity framework may help social and public policy actors to avoid major challenges and problems. These arguments as discussed about the polarised views and practices on policy processes, compels to explore the possibilities manoeuvre the middle way as presented below ( Figure 5).

The proposed middle way as presented in the Figure 5 circles around the dialogical progressive centre. This dialogical progressive centre relates to all the constructs around the polarised ends. The middle way process relates to the argumentative and critical end through the instruments of realistic reasoning and consultative, engaging approaches. The argumentative and critical end is stimulated by ideological constructs, such as left and south, while the neo-managerial and administrative end is stimulated by ideological constructs, such as right and north. The argumentative and critical end is complemented by participatory and consensus approaches that emerge through social responsiveness and extreme rationalism. The middle way process relates to the neo-managerial and administrative end through the instruments of pragmatic strategies and result orientation. The neo-managerial and administrative end is complemented by technocratic and cost-benefit analysis, which results through economic responsiveness and extreme empiricism. Thus, the middle way of the dialogical and progressive educational policy framework aims to engage a wide range of ideas, ideologies, actors, processes, and systems. The middle way is not a perfect space but a potential space in which a dialogical and progressive policy making may thrive.

## Conclusion

The study aimed to manoeuvre a middle way to propose a dialogical and progressive educational policy framework amidst the polarised constructs. The first three phases of Lynham’s (2002) theory building such as conceptual development, operationalisation and confirmation/disconfirmation helped to explore answers to the research question of the study: ‘how could a middle way (a dialogical and progressive framework) be manoeuvred among the polarised (new-liberal/managerial vs argumentative/critical frameworks) policy constructs? The first three phases and the exploration of relevant theories, models and related literature helped the study to content that if it is possible to have a polarised neo-managerial, market oriented and critical, argumentative policy frameworks, it should be then possible to have a dialogical, progressive middle way policy framework. The fourth phases ‘application’ and the fifth phase ‘continuous refinement’ could be explored by future research on the proposed dialogical progressive educational policy framework. As a theory is never complete, it is important that the theory be continually refined and developed ( Rensburg, Motala & David, 2015b). The outcome of the application of the framework will help to refine and further develop this dialogical progressive policy framework. Every research and every research method have their own limitations. Lynham (2002) acknowledges that this five-phase theory building method in applied disciplines is much less programmatic and the method could be further refined by theorists, however, that is not the aim of this research. As this research aims to develop a dialogical progressive policy framework, it mainly focused on developing, operationalising, confirming, and disconfirming, of this dialogical progressive policy framework. The study had to make careful choices of the theories, models and approaches that spans the public and educational policy spectrum, however, the research had to limit to the most important theories, models which possibly impacted the exclusion of other relevant theories and models. Future refinement of this framework could address such limitations. As Peters (2005) points out that the instruments used to address policy problems have much less developed conceptions of those problems themselves and as Dryzek (2008) indicates that policy processes as an ongoing dialogue and critique, the proposed ‘dialogical and progressive policy framework’ would evolve further depending on the constructive critiques and dialogues. The research is open for any such constructive critiques to further this framework and strongly believes that the policy science and practice might continue to evolve with much openness. The dialogical and progressive (middle way) educational policy framework aims to engage a wide range of ideas, ideologies, actors, processes, and systems. The study believes that the proposed middle way is not a perfect space but a potential space in which a dialogical and progressive educational policy may thrive.

## Data Availability

No data are associated with this article.
