# Supplementary material for: Towards a dialogical and progressive educational policy framework: Manoeuvring a middle way among the polarised constructs
Source: F1000Res. 2023 May 26;12:74. Originally published 2023 Jan 19. [Version 2] doi: 10.12688/f1000research.127762.2 (PMC10285327; doi:10.12688/f1000research.127762.2)
Supplement: Peer review history

# Peer review: Reviewer response for version 2

**DOI:** 10.5256/f1000research.147776.r175436

**Published:** 2023-06-13

**Authors:** Terry Wotherspoon

**Affiliations:** 1 University of Saskatchewan, Saskatchewan, Canada

The revised article addresses the main comments highlighted in the review, resulting in a much more focused and improved manuscript.

Is the work clearly and accurately presented and does it cite the current literature?

Partly

If applicable, is the statistical analysis and its interpretation appropriate?

Not applicable

Are all the source data underlying the results available to ensure full reproducibility?

No source data required

Is the study design appropriate and is the work technically sound?

Yes

Are the conclusions drawn adequately supported by the results?

Partly

Are sufficient details of methods and analysis provided to allow replication by others?

No

Reviewer Expertise:

sociology of education; social policy analysis; social inequality

I confirm that I have read this submission and believe that I have an appropriate level of expertise to confirm that it is of an acceptable scientific standard.

# Peer review: Reviewer response for version 2

**DOI:** 10.5256/f1000research.147776.r175435

**Published:** 2023-06-02

**Authors:** Hugo Horta

**Affiliations:** 1 Social Contexts and Policies of Education, Faculty of Education, The University of Hong Kong, Hong Kong, China

I believe the revised manuscript is stronger and can be indexed.

Is the work clearly and accurately presented and does it cite the current literature?

Partly

If applicable, is the statistical analysis and its interpretation appropriate?

Not applicable

Are all the source data underlying the results available to ensure full reproducibility?

No source data required

Is the study design appropriate and is the work technically sound?

Partly

Are the conclusions drawn adequately supported by the results?

Yes

Are sufficient details of methods and analysis provided to allow replication by others?

Yes

Reviewer Expertise:

Science and public policy; Higher Education

I confirm that I have read this submission and believe that I have an appropriate level of expertise to confirm that it is of an acceptable scientific standard.

# Peer review: Reviewer response for version 1

**DOI:** 10.5256/f1000research.140302.r169854

**Published:** 2023-05-02

**Authors:** Terry Wotherspoon

**Affiliations:** 1 University of Saskatchewan, Saskatchewan, Canada

This paper provides a potentially useful contribution to a theoretical approach to social policy, oriented to guide what the author poses as “a dialogical and progressive educational policy framework.” In addressing the central question of how a middle way for policy construction and formulation be navigated within the current (educational) policy environment, the discussion covers an extensive range of relevant policy literature and themes.

 While the aims of the paper are worthwhile exploring, the discussion could benefit from considerable tightening of its scope and focus in order to offer a more compelling case. In particular, it would be useful to provide readers with a stronger sense of why or how this question matters, or what the main focus is in advancing such a question. This clarify of focus would offer a clearer understanding of the relationship between policy analysis (theoretical approaches oriented to the understanding or explanation of policy-related matters) and policy processes (stages in the framing and implementation of policy, which the paper seeks to inform or influence); the paper in its current form blurs this distinction as it moves repeatedly back and forth from one to the other. The discussion is positioned as a form of theory-building and ideological investigation, and therefore focuses on the more abstract dimensions associated with the former, but in trying to make the case that a middle way position should be implemented, it also highlights more substantive processes associated with the latter. In the process, the paper loses some of its potential impact by trying to cover too many disparate themes and bodies of literature while leaving aside important points of clarification and elaboration most pertinent to the intended focus. Four main sets of revisions are suggested:

1. Although the paper makes clear that its focus is not empirical, it addresses matters that require at least some attention to actual factors that drive or influence policy directions and policy-related decision-making. While drawing from literature that highlights the managerial and neo-liberal influences on current policy frameworks, current educational policy frameworks are in fact highly complex in nature; it should not be assumed that educational policies are fully coherent, nor that they are influenced by any single policy orientation. The literature on path dependence (e.g., P. Pierson) demonstrates the barriers governments have encountered even if strongly committed to neoliberal policy directions, while critical analysis of education (e.g., M. Apple, M. Carnoy and H.M. Levin) highlights ongoing tensions and contradictions in the formation of educational policies. The paper aims “to stimulate dialogue among polarised frameworks” but, of course it is not approaches but actors who engage in policy dialogue, employing particular discourses derived not only from various ideological or political stances, but also on practical considerations including resources and governance structures. How, in other words, or at what level, would the anticipated dialogue be conducted? Surprisingly, the paper makes no reference to literature in which such a dialogue has actually been initiated – the ‘third way’ approach advanced initially in the work of Anthony Giddens, as well as broader ‘social investment’ policy orientations in which education features centrally.
2. The discussion would be strengthened by the inclusion of a bit more detail or specificity in the discussion of the methodology employed in the analysis. While described, variously, as a rationalist and hermeneutic approach, applied through discourse analysis, these terms and how they are understood by the author are not defined explicitly in the paper. A brief statement in this regard, along with further information as to how they were actually employed in the analysis, would be useful. This would provide the reader with further information about how decisions were made to classify certain statements, thereby helping to justify more fully some of the main points or conclusions presented in the paper.
3. In many places discussion that is somewhat superfluous to the main focus could be removed. Some of the sections (in particular the section on Operationalization, but in other places as well) include discussions presented as distinct literature reviews, making reference to disparate themes, concepts, and authors which are not fully integrated with one another and which appear to be somewhat secondary to the main analysis. It would be possible to remove some of this material, thereby enhancing or concentrating attention to the main points within the analysis while making space to address the revisions proposed in the previous two points.
4. Careful proofreading is recommended in order to address minor editorial points and clarify some a few ambiguous statements.

Is the work clearly and accurately presented and does it cite the current literature?

Partly

If applicable, is the statistical analysis and its interpretation appropriate?

Not applicable

Are all the source data underlying the results available to ensure full reproducibility?

No source data required

Is the study design appropriate and is the work technically sound?

Yes

Are the conclusions drawn adequately supported by the results?

Partly

Are sufficient details of methods and analysis provided to allow replication by others?

No

Reviewer Expertise:

sociology of education; social policy analysis; social inequality

I confirm that I have read this submission and believe that I have an appropriate level of expertise to confirm that it is of an acceptable scientific standard, however I have significant reservations, as outlined above.

# Peer review: Reviewer response for version 1

**DOI:** 10.5256/f1000research.140302.r166893

**Published:** 2023-03-30

**Authors:** Hugo Horta

**Affiliations:** 1 Social Contexts and Policies of Education, Faculty of Education, The University of Hong Kong, Hong Kong, China

The paper offers a relevant contribution to the advancement of knowledge to the field, and I believe it only needs some minor revisions before it can be indexed. I offer below, my suggestions for the improvement of the paper:

  

-  Although I understand the standpoint of the author, it may be a bit too bold to say that this is the first study trying to complement different views into one that assumes a more middle ground stance. A more moderate stance would be welcome. One also has the impression from the text that education policies tend to be essentially polarized, when in fact most of the times what happens is a constant balancing act and dialogue between polarizing policy and related ideological views, and often views that we believe to be dominant are not as dominant as we consider them to be. The paper by Capano and Pritoni ^1^ proves this point for example and should be taken into account.

 

- More information about the literature review would also be helpful. For example, the author may want to be detailed in his search for relevant literature. I would be expecting to see some keyword searching in databases such as Scopus and the Web of Science, and an analysis of policy and policy topics in specialized education levels. For example, in higher education, there is a journal that is essentially focused on policy (Higher Education Policy) and the broad journals such as Higher Education and Studies in Higher Education often analyze policy, values, ideology and the public/private dialectic in the field. It may be worthwhile to have a look at some of these journals.

 

- There are two spheres that I have not seen so present in the paper and that I would have liked to. One of them is the policy process (including the design stage) in its relevance with uncertainty (what some authors call illities) and understand the parallels between those policies that cover safer and certain topics (thus more conservative and usually supported by international organizations) and those policies that assume a bolder perspective because they are dealing with new topics, that attempt to meet policy goals that are uncertain and in a set dominated by overall uncertainty. The other sphere that I would have liked to see more evident in the paper, is the issue of culture, which is key in mediating/moderating the design (and implementation) of policies in different countries and settings. The very idea of public and corporate interest – as an example – can be strongly shaped by the cultural dimensions of a government, country, ethnic group, region, etc. In a way, culture works as a balancing/moderating factor between different perspectives that seem polarizing. Essentially, more debate, conceptualization and reflection on these issues should be included in the current version of the paper to add further value to it.

Is the work clearly and accurately presented and does it cite the current literature?

Partly

If applicable, is the statistical analysis and its interpretation appropriate?

Not applicable

Are all the source data underlying the results available to ensure full reproducibility?

No source data required

Is the study design appropriate and is the work technically sound?

Partly

Are the conclusions drawn adequately supported by the results?

Yes

Are sufficient details of methods and analysis provided to allow replication by others?

Yes

Reviewer Expertise:

Science and public policy; Higher Education

I confirm that I have read this submission and believe that I have an appropriate level of expertise to confirm that it is of an acceptable scientific standard, however I have significant reservations, as outlined above.

## References

[1] : What really happens in higher education governance? Trajectories of adopted policy instruments in higher education over time in 16 European countries. *Higher Education* .2020;80(5) : 10.1007/s10734-020-00529-y 989-1010 10.1007/s10734-020-00529-y
